# Targeting Neutrophil Extracellular Traps: A New Strategy for the Treatment of Acute Ischemic Stroke Based on Thrombolysis Resistance

**DOI:** 10.1055/a-2609-3457

**Published:** 2025-06-03

**Authors:** Genquan Huang, Hao Wu, Bintong Lin, Dezhi Deng, Yuan Liu, Juan Qu, Junjie Xu, Baoxiang Wang

**Affiliations:** 1 Jiaxing University Master Degree Cultivation Base, Zhejiang Chinese Medical University, Hangzhou, People's Republic of China; 2 Department of Neurology, Affiliated Hospital of Jiaxing University, The First Hospital of Jiaxing, Jiaxing, People's Republic of China; 3 Department of Neurology, Xi'an Traditional Chinese Medicine Hospital, Xi'an, People's Republic of China

**Keywords:** neutrophil extracellular traps, acute ischemic stroke, thrombolysis resistance, deoxyribonuclease I, intravenous thrombolysis

## Abstract

Acute ischemic stroke (AIS) is a life-threatening thrombotic disorder, with intravenous thrombolysis (IVT) serving as the first-line treatment during its acute phase. However, thrombolysis resistance diminishes the success rate of early reperfusion. Recent studies have highlighted neutrophil extracellular traps (NETs) as a critical factor contributing to thrombolysis resistance. Targeting NETs with deoxyribonuclease I (DNase I) has been shown to significantly improve the thrombolytic efficacy of recombinant tissue plasminogen activator (rt-PA) and reduce the risk of hemorrhagic transformation. In this review, we summarize current knowledge on the mechanisms by which NETs contribute to thrombosis and thrombolysis resistance, explore the prospective and feasibility of targeting NETs to improve thrombolysis, providing information about the creation of innovative thrombolytic treatment approaches for AIS.

A class of cerebrovascular illnesses known as strokes has a high prevalence of disability and death. Stroke is the third most common cause of mortality and the fourth most common source of disease burden worldwide, according to the most recent statistics from the Global Burden of Disease Study. 1 2 Among various types, the most frequent kind is ischemic stroke. 3 As an arterial thromboembolic event, early reperfusion is essential for acute ischemic stroke (AIS) treatment to preserve the ischemic penumbra; the sole FDA-approved thrombolytic medication for AIS at the moment is recombinant tissue plasminogen activator (rt-PA). 4 However, its therapeutic efficacy is significantly limited by a narrow therapeutic time window and numerous contraindications, with less than 15% of new-onset AIS patients receiving intravenous thrombolysis (IVT) in most European countries. 5

More concerning, the early recanalization rate with rt-PA is only about 33%. 6 Although rt-PA continues to dissolve fibrin, platelets may still be recruited to the dissolved thrombus, potentially leading to secondary occlusion. 7 This phenomenon, known as thrombolysis resistance, was initially attributed to factors such as thrombus location, 6 thrombus length, 8 post-translational modification of fibrin, 9 and clot contraction. 10 However, recent advancements in mechanical thrombectomy (MT) have provided technical support for acquiring pathological thrombi, enabling deeper analysis of thrombus composition and structure. This research has revealed that non-fibrin components, such as neutrophil extracellular traps (NETs), leukocytes, and von Willebrand factor (vWF), are also critical contributors to thrombolysis resistance. 11 12 13

Neutrophils are the first to be drawn to the infection site and are crucial protectors of the host's innate immune response, where they perform immune defense functions through three primary mechanisms: phagocytosis of pathogens, release of antimicrobial granules, and formation of NETs. 14 Beyond their role in infectious diseases, neutrophils are also a part of the aseptic inflammation that occurs when tissue is damaged. 15 Studies have shown that neutrophils extravasate from the leptomeningeal vessels after stroke and gradually accumulate at the lesion site. Notably, neutrophils show indications of activity, such as histone H3 citrullination, chromatin decondensation, and the release of cellular contents indicative of NETs formation. 16 17 NETs have been found in AIS patients' plasma and thrombi, and their concentrations are highly connected with the severity of the illness and a bad prognosis. 18 19 20 Further research has demonstrated that NETs contribute to thrombosis through mechanisms such as platelet interaction, 21 promotion of atherosclerosis, 22 and activation of the coagulation cascade. 23 Additionally, NETs provide scaffolds for platelet and erythrocyte aggregation, 24 alter the fibrin architecture, and form a hard thrombus shell, 25 which acts as a barrier to thrombolysis.

The processes of NETs generation and their function in AIS will be covered in this review, with a focus on their involvement in thrombolysis resistance. We will also explore the potential of targeting NETs as an adjunctive strategy in AIS thrombolytic therapy.

## Basics of NETs

### Origins of NETs

In 2004, Brinkmann et al first observed neutrophils forming extracellular network structures under an electron microscope after stimulation with interleukin-8, phorbol myristate acetate (PMA), or lipopolysaccharide. These activated neutrophils released histones, DNA, and granule proteins—such as neutrophil elastase (NE), myeloperoxidase (MPO), and cathepsin G (CG)—which captured and killed bacteria. This structure was named “NETs,” and “NETosis” was the name given to the process by which they formed. 26 Due to characteristic chromatin decondensation and the inability of both NADPH oxidase (NOX) inhibitor-treated neutrophils as well as neutrophils from chronic granulomatous disease patients (which involves a defective NOX gene) to form NETs under PMA stimulation, 27 NETosis was initially considered a NOX-dependent process distinct from apoptosis and necrosis. 28 Further studies have demonstrated that a range of stimuli binding to distinct receptors can cause NETosis, including bacteria and their derivatives, 29 30 31 32 fungi, 33 viruses, 34 immune complexes, 35 specific cytokines (e.g., IL-1β, IL-8, and TNF-α), 36 and crystals. 37

### Formation Mechanism of NETs

Suicidal NETosis is the first identified and most studied form of NETs formation. It is characterized by its dependence on NOX and is accompanied by neutrophil death. Using the classical stimulus PMA as an example, activation of neutrophil surface receptors increases intracellular calcium levels, which activates protein kinase C (PKC) and peptidylarginine deiminase 4 (PAD4). This cascade subsequently induces the generation of reactive oxygen species (ROS) from NOX2 via the Raf/MEK/ERK signaling pathway. 38 ROS then triggers the dissociation of NE from the azurophilic granule complex into the cytoplasm, where F-actin is degraded as a result of MPO activating NE's proteolytic action. After that, NE moves into the nucleus, where it breaks down histones and aids in chromatin decondensation. 39 40 PAD4 is essential to the chromatin decondensation mechanism described above, and one of the possible mechanisms is that PAD4 reduces the positive charge on the histone surface by catalyzing the citrullination of histones, thereby weakening its electrostatic interaction with DNA. 41 42 Ultimately, a pore-forming protein, gasdermin D (GSDMD), creates holes inside the plasma and nuclear membranes, permitting citrullinated histones to be released, DNA and released proteins in the extracellular space such as granules come together to form NETs. 31 43

The second type, termed vital NETosis, is characterized by independence from NOX, and neutrophils always maintain plasma membrane integrity and functional activity. This process has been shown to be triggered by *Staphylococcus aureus* or Gram-negative bacteria via Toll-like receptors (TLRs) and complement receptors. 44 45 *Staphylococcus aureus* , a highly invasive pathogen, is rapidly captured by neutrophils, which form NETs to contain its spread. Upon stimulation of Toll-like receptor 2 (TLR2) and complement receptors by *Staphylococcus aureus* , neutrophils undergo rapid changes: their multilobular nuclei round-up and concentrate, and the nuclear membranes' inner and outer layers split apart. Nuclear DNA and granule proteins sprout from the outer nuclear membrane and form vesicles, which are exocytosed onto the plasma membrane and ultimately assembled into NETs in the extracellular space. The remaining neutrophil nucleus, though empty, retains its defensive role. 32 46

Mitochondrial NETosis, first identified and described by Yousefi et al, occurs after stimulation with granulocyte-macrophage colony-stimulating factor (GM-CSF), lipopolysaccharide (LPS), or complement factor 5a. In this process, neutrophils release DNA from mitochondria in a way that is dependent on ROS to form NETs without inducing neutrophil death. 47 Recent studies have shown that the mechanism of mitochondrial ROS and NETs formation involves the opening of the mitochondrial permeability transition pore. 48

## Pathologic Role of NETs in AIS

### Kinetics of NETs during AIS

Following the onset of acute ischemic stroke (AIS), the hypothalamic–pituitary–adrenal (HPA) axis and sympathetic nervous system are rapidly activated. Glial cells and damaged neurons release damage-associated molecular patterns (DAMPs), which induce endothelial cells to express chemokines. As a result, neutrophils are mobilized from the bone marrow and spleen into the peripheral circulation. These circulating neutrophils then undergo a well-orchestrated sequence of events, including intravascular rolling, adhesion, transendothelial migration, and ultimately infiltration into the brain parenchyma. 49

In the absence of reperfusion, peripheral neutrophils predominantly access the brain via leptomeningeal vessels, traversing along the Virchow–Robin space into the perivascular space, and subsequently penetrating the brain parenchyma. In fact, neutrophils are already activated prior to their entry into the brain parenchyma. 17 Notably, neutrophil infiltration into the brain occurs in a time-dependent manner, suggesting that the rate of infiltration is closely associated with the extent of basement membrane disruption. 50

Based on the synthesis of relevant studies ( Table 1 ), we preliminarily propose the following timeline: NETs begin to form intravascularly within 0.5 to 6 hours post-stroke, infiltrate the brain parenchyma between 6 and 24 hours, and peak at 2 to 5 days. However, there is considerable variability among the findings of different studies. We attribute this variability to several factors: (1) Differences in animal species and experimental models, notably, neutrophil infiltration appears to be more pronounced following pMCAO compared with tMCAO. 51 (2) Variability in the sensitivity of detection methods. (3) Some studies initiated measurements too late and employed prolonged intervals between time points, lacking continuous early-phase dynamic monitoring. 52 53 54 55

**Table 1:** Compilation of NETs kinetic studies during AIS

Animals	Model	Methods	Viewpoints	Note	Reference
ICR mice	Carotid occlusion	IF	The count of NETs within thrombi began to increase starting at 0.5 hour after occlusion	None	52
WT C57BL/6 mice	tMCAO	QFM	NETs formed in cerebral vasculature by 6 hours post-stroke, spread to brain parenchyma by 12 hours, and peaked at 24 hours	None	53
SD rats	pMCAO	IF, WB	NETs enter via the leptomeninges at 6 hours post-stroke, appear in the cortex and peripheral blood by 12 hours, and reach the striatum by 24 hours	The earliest detection time points were set at 6 hours for vasculature and 12 hours for the parenchyma	54
WT C57BL/6 mice	pMCAO	WB	NETs appear in the ischemic cortex by 1 day post-infarction, peaking at 3–5 days	The detection time points spanned days 1, 3, and 5	55
Balb/C mice	pMCAO	IF	NETs can be detected in the capillary lumen, perivascular space, and parenchyma before 24 hours after ischemia	Lack of specific early detection time points	17
WT C57BL/6 mice	tMCAO	FM, SEM	NETs formed in the cortex by 6 hours and striatum by 12 hours post-stroke, peaking at 2–3 days	The earliest detection time point was at 6 hours	16

Abbreviations: FM, fluorescence microscopy; IF, immunofluorescence; pMCAO, permanent middle cerebral occlusion; QFM, quantitative fluorescence microscopy; SEM, scanning electron microscope; tMCAO, transient middle cerebral artery occlusion; WB, Western blot.

### Involvement of NETs in Thrombosis

An increasing body of research has shown that innate immune cells, including neutrophils and monocytes, contribute to immune defense by participating in thrombosis. In 2013, this physiological process was referred to as immunothrombosis by Engelmann and Massberg. 56 Although immunothrombosis helps to limit the spread of pathogens, its dysregulation can lead to thrombotic diseases, including cerebral infarction, myocardial infarction, and deep vein thrombosis. 57 The discovery of NETs in both venous and arterial thrombosis offers direct evidence of their involvement in these processes. 58 59 Laridan et al analyzed thrombi taken from AIS patients undergoing MT. They found that neutrophils were found in every thrombi, and the existence of NETs was shown by the colocalization of citrullinated histone H3 (CitH3) with extracellular DNA. 60 Similarly, Ducroux et al demonstrated that 108 AIS thrombi samples had NETs present in large quantities, primarily localized to the outer layer of the thrombus. 61 Importantly, the content of NETs in thrombi has been identified as a potential predictor of AIS severity and poor functional outcomes. 19 20

#### NETs Interact with Platelets to Promote Thrombosis

Under normal physiological conditions, neutrophils and platelets do not interfere with each other. However, in pathological states, their interaction leads to mutual activation, influencing both NETs formation and thrombus development. 21 62 Upon platelet activation, P-selectin and CD40 ligand (CD40L) expression on the platelet surface is upregulated. 63 These molecules then bind to P-selectin glycoprotein ligand-1 (PSGL-1) and CD40 on neutrophils, respectively, activating macrophage-1 antigen (Mac-1) through a tyrosine kinase-dependent mechanism. 64 Once activated, Mac-1 binds to platelets via GPIbα and intercellular adhesion molecule 2 (ICAM2), promoting neutrophil–platelet adhesion. 65 66 Additionally, activated platelets may promote the recruitment and activation of neutrophils by releasing serotonin, chemokines, and high mobility group box-1 protein (HMGB1). 67 68 69 Conversely, activated neutrophils promote platelet activation and thrombosis through the release of cathelicidins and NETs. 70 71 72 73 Fuchs et al induced neutrophils to form NETs using PMA and subsequently perfused the NETs with platelets. Under electron microscopy, they observed time-dependent platelet aggregation on the NETs, along with platelet activation. 24 Then, they perfused the NETs with blood either treated or untreated with deoxyribonuclease I (DNase I). In the DNase I-treated group, NETs were rapidly degraded and platelet aggregates did not form. After 10 minutes, DNase I was added to the untreated group, resulting in the rapid clearance of both NETs and platelet aggregates. These findings suggest that NETs serve as scaffolds for platelet aggregation.

Recent studies highlight HMGB1 as a key mediator of the crosstalk between NETosis and thrombosis: during the acute phase of cerebral ischemia, HMGB1 expression on platelets is significantly upregulated, mediating platelet aggregation, activation, and thrombosis via the TLR4/MyD88 and cGMP/PKG pathways. 74 75 Activated platelets release HMGB1, it subsequently attaches to neutrophils' TLR4 and receptor for advanced glycation end products (RAGE), causing NETosis and encouraging thrombus development. 67 75 76 77

#### NETs Promote the Coagulation Cascade

NETs can promote the coagulation cascade through several mechanisms. Key processes include NETs components enhancing thrombin production by activating platelets, triggering endogenous coagulation pathways, and degrading inhibitors of exogenous coagulation pathways. 23 Extracellular histones can induce platelet activation and procoagulant phenotype expression through TLR2 and TLR4, which in turn promote the generation of plasma thrombin. The presence of DNA further enhances histones' ability to stimulate thrombin production. 71 72 Additionally, NETs' negatively charged DNA backbone can bind and activate factor XII, thereby enhancing the endogenous coagulation pathways and shortening clotting time when DNA is added to human plasma. 78 79 Interestingly, Noubouossie et al shown that coagulation activation is not directly promoted by intact NETs in vitro, which may be due to interactions between histones and DNA within the nucleosome that neutralize the negative charge of the NETs' DNA surface. 80 Furthermore, NE and CG in NETs can inactivate tissue factor pathway inhibitor (TFPI), thereby enhancing the tissue factor (TF)-induced coagulation pathway. 81 Zhou et al demonstrated that NETs contribute to the hypercoagulability in AIS patients with internal carotid artery occlusion. They found phosphatidylserine (PS)-bearing NETs in the plasma and thrombi of these patients, which enhanced platelet aggregation and coagulation factor deposition, promoting thrombin and fibrin formation. NETs-derived proteases and histones also exert toxic effects on vascular endothelial cells (ECs), inducing a procoagulant phenotype in ECs by promoting PS exposure and TF expression. 82 83 84

### NETs Involved in Atherosclerosis

Arterial occlusion caused by atherosclerotic thrombus is a significant cause of ischemic stroke. 85 Recent research has revealed that NETs exist in atherosclerotic plaques. 86 In ApoE knockout (Apoe −/−) mice fed a high-fat diet for 3 weeks, NETs were detected in the atherosclerotic lesions of the aortic root. 87 Blocking NETs formation in this model by injecting Cl-amine, a peptidylarginine deiminase (PAD) inhibitor, reduced the formation of carotid atherosclerotic plaques. 88 Liu et al constructed PAD4 gene knockout mice, which further confirmed that the specific deletion of PAD4 reduced NET formation and vascular inflammation and caused a significant reduction in atherosclerotic burden in Apoe−/− mice. 87 However, Franck et al found that while PAD4 gene defects reduced EC damage and plaque erosion by inhibiting NETosis, they did not significantly impact atherosclerosis formation or progression in hypercholesterolemic mice. 89

NETs may contribute to atherosclerosis through two primary mechanisms: inflammatory stimulation and immune activation, with oxidized low-density lipoprotein (ox-LDL) and cholesterol crystallization playing key roles. First, NETs can directly induce EC death, leading to collagen exposure and platelet aggregation, which further triggers NETosis and exacerbates local inflammation, creating a vicious cycle that promotes atherosclerosis. 90 91 On the one hand, MPO can induce the oxidative modification of LDL, and ox-LDL is subsequently phagocytosed by macrophages and forms foam cells, which contribute to the formation of atherosclerotic plaque by accumulating underneath the artery intima. 92 93 Conversely, ox-LDL can activate TLR-PKC-IRAK-MAPK and NADPH oxidase pathways, stimulating the NETs generation and aggravating chronic inflammation in the arterial intima. 94 Additionally, cholesterol crystal-induced NETs can trigger the release of IL-1β by macrophages, which in turn activates T helper 17 (Th17) cells and upregulates the expression of IL-17. This cascade amplifies immune cell recruitment to the lesion site, promotes vascular inflammation and endothelial dysfunction, and ultimately increases the development and spread of atherosclerotic plaques. 95 Recent studies also show that low shear stress, resulting from hemodynamic changes, can induce NETs formation via Piezo1, a mechanically gated ion channel, thereby exacerbating atherosclerosis. 96

## NETs and Thrombolysis Resistance

### NETs as Thrombus Components Affect Thrombolytic Efficacy

IVT has offered hope for reperfusion therapy of AIS, but it is not equally effective against all thrombi, particularly in cases of cardioembolic stroke. 97 The composition and structure of thrombus are now considered to be the critical factors influencing thrombolytic efficacy. 98 Although AIS thrombi exhibit considerable heterogeneity, they can generally be categorized into erythrocyte-rich and platelet-rich regions. The erythrocyte-rich regions typically have a simpler structure, primarily consisting of erythrocytes embedded in a thin fibrin network. Conversely, areas that are rich in platelets are distinguished by a thick network of fibrin that acts as a scaffold, along with non-fibrin components such as vWF, leukocytes, extracellular DNA, or NETs. NETs are often localized along the thrombus surface, especially in places that are rich in platelets or at the intersection of regions that are rich in erythrocytes and platelets. 12 99 100 Numerous studies have shown that cardioembolic thrombi, compared with atherosclerotic thrombi, have a higher fibrin/platelet ratio, contain more leukocytes and NETs, and have fewer erythrocytes. 101 102 103 This compositional difference may help explain the variability in thrombolytic efficacy. In vitro experiments have demonstrated that erythrocyte-rich thrombi are more susceptible to rt-PA than thrombi that are rich in platelets, a finding that aligns with clinical observations. 98 104

### Mechanism of NETs Leading to Thrombolysis Resistance

Fuchs et al's study further demonstrated that, beyond activating platelets and serving as scaffolds for platelet and erythrocyte aggregation, NETs also promote fibrin formation and deposition by interacting with vWF, fibronectin, and fibrinogen, thereby enhancing thrombus stability. 24 They also compared the sensitivity of NETs and fibrin to thrombolysis in in vitro clots. It was found that rt-PA could effectively remove fibrin but could not prevent subsequent thrombus formation. In rt-PA-resistant clots, platelets and erythrocytes remained bound together via the DNA scaffold of NETs. Only when rt-PA was used in combination with DNase I could thrombus formation be inhibited. Therefore, NETs can provide a thrombus scaffold that is independent of fibrin and resistant to rt-PA.

**Fig. 1:**
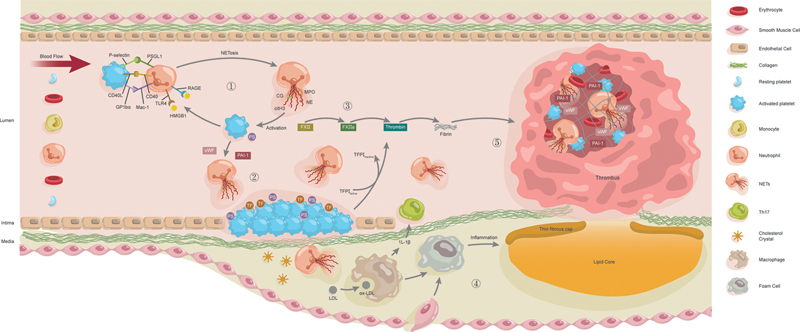
Mechanisms of neutrophil extracellular traps (NETs) involved in rt-PA-resistant thrombosis. ① Platelets and neutrophils interact through specific ligands and receptors, leading to mutual activation. High mobility group box-1 protein (HMGB1) released by activated platelets binds to the receptor for advanced glycation end products (RAGE) on neutrophils, inducing NETosis. ② NETs damage endothelial cells (Ecs), thereby inducing Ecs to expose collagen and phosphatidylserine (PS) and express tissue factors (TF), which cause platelet aggregation and procoagulant phenotype expression. ③ Activated platelets with a procoagulant phenotype, along with the DNA backbone of NETs, activate factor XII (FXII), initiating the endogenous coagulation pathway. NETs further enhance coagulation by inactivating tissue factor pathway inhibitor (TFPI). ④ Macrophages engulf NETs-induced oxidized low-density lipoprotein (ox-LDL) to form foam cells and develop into atherosclerotic plaques, and NETs aggravate inflammation and endothelial dysfunction by initiating IL-1β/Th17, resulting in unstable plaque rupture and arterial thrombosis. ⑤ NETs provide scaffolds for platelet and erythrocyte aggregation and promote fibrin fiber thickening, thus enhancing thrombus stability. Additionally, NETs stimulate the release of von Willebrand factor (vWF) and PAI-1 from platelets and ECs. Together with the platelets and NETs, this forms a dense shell together with NETs and platelets, ultimately exacerbating thrombolysis resistance (created with Adobe illustrator).

As the primary constituents of NETs, histones, and extracellular DNA can alter the fibrin architecture in thrombi and make them thicker fibrin fibers, accompanied by lower permeability, they can exert anti-fibrinolytic effects. 105 106 Zhang et al found that NETs contribute to blood hypercoagulability, leading to microthrombosis and consumption of rt-PA. Importantly, NETs make it easier for PS to reach platelet and EC surfaces, enhancing their procoagulant activity and promoting the release of vWF and plasminogen activator inhibitor-1 (PAI-1), which further contribute to thrombolysis resistance. 107 Di Meglio et al examined the thrombi of 199 AIS patients with large vessel occlusion using scanning electron microscopy and immunohistochemistry. They observed that all these thrombi exhibited a shell composed of dense thrombus components (including fibrin, vWF, platelets, extracellular DNA, and NETs), which formed a barrier that hindered thrombus dissolution by rt-PA. 25 One of the mechanisms may be that the NETs in the shell limit the binding of rt-PA to its substrate (fibrin). Recent proteomic studies support these findings, showing that the amount of fibrin(ogen) within AIS thrombi correlates positively with the presence of NETs. The dense fibrin cap created by this interaction reduces fibrinolytic activity by decreasing thrombus permeability. Furthermore, the large number of neutrophils and NETs surrounding the thrombus impairs fibrinolysis by covering the thrombus surface, ultimately contributing to thrombolysis resistance. 108 The current knowledge of NETs involved in rt-PA-resistant thrombosis is summarized in Fig. 1 .

## Current Strategies for Improving Thrombolysis Resistance

Although MT is a valuable treatment, it remains limited in availability, making IVT the preferred reperfusion therapy for AIS in most regions. Therefore, improving the effectiveness of IVT remains of critical clinical importance. The composition of an AIS thrombus is highly complex, and since rt-PA primarily targets fibrin, its “one-size-fits-all” approach is insufficient to achieve optimal recanalization. Recent trials targeting non-fibrin components of AIS thrombus have provided valuable insights into overcoming thrombolysis resistance ( Table 2 ).

**Table 2:** Summary of potential drug targets for improving thrombolysis resistance

Target	Classification	Drugs	Mechanisms	Clinical trials
NETs	DNA degrader	Dornase Alfa	Recombinant human form of DNAse 1	NETS-target, NCT04785066; EXTEND-IA DNase, NCT05203224
	PAD4 inhibitor	GSK484	Inhibits NETs formation and thrombosis	None
		GSK199	Reduces NETs formation and infarct volume	None
		nNIF	Reduces NETs formation and infarct volume	None
	ROS inhibitor	Vitamin C	Reduces NETs formation	PSIOM, NCT03543917
		Edaravone	Reduces BBB damage	TASPE, NCT06248242
vWF	vWF degrader	N-acetylcysteine	Cleaves disulfide bonds within vWF polymers	NAC-S, NCT04920448; NCT04918719
		ADAMTS13	Specific VWF-cleaving metalloprotease	NCT02219035
	RNA aptamer	BB-031	Inhibits vWF activity	RAISE, NCT06226805
Platelet	GPIIb/IIIa inhibitor	Eptifibatide	Prevents platelet aggregation	CLEAR-ER, NCT00894803; CLEAR-FDR, NCT01977456
	Monoclonal antibody of GPVI	Glenzocimab	Inhibits platelet adhesion	ACTISAVE, NCT05070260; GALICE, NCT06437431
	P2Y12 inhibitor	Cangrelor	Inhibits platelet aggregation and activation	REPERFUSE, NCT04667078
Fibrin	TAFI inhibitor	DS-1040b	Promotes fibrinolysis	ASSENT, NCT02586233; NCT03198715
	PAI-1 modulator	THR-18	Promotes fibrinolysis	NCT01957774; NCT02572336
	Monoclonal antibody of α2-AP	TS23	Inhibits endogenous plasminogen activator	SISTER, NCT05948566

Abbreviations: BBB, blood–brain barrier; NETs, neutrophil extracellular traps; PAI-1, plasminogen activator inhibitor-1; TAFI, thrombin activatable fibrinolysis inhibitor; vWF, von Willebrand factor; α2-AP, α2-antiplasmin.

### Strategies Targeting NETs

Therapeutic strategies targeting NETs mainly involve inhibiting NETs formation or promoting their degradation. The activation of PAD4 and the generation of ROS are central to NETosis. Thus, inhibiting PAD4 and ROS production is key to suppressing NETs formation. GSK484, a specific and reversible PAD4 inhibitor, has been shown to inhibit thrombosis and alleviate inflammatory damage in preclinical models of subarachnoid hemorrhage by blocking NETs formation. 109 110 Moreover, neonatal NET-inhibitory factor (nNIF) and GSK199, new PAD4 inhibitors, have been demonstrated to reduce NETs levels in the plasma of tMCAO mouse models and significantly decrease infarct volume, thereby improving stroke outcomes. 111 In the context of AIS, oxygen radical scavengers that target ROS, such as vitamin C and edaravone, have shown promise in inhibiting NETs formation. 112 A recent meta-analysis demonstrated that vitamin C intervention reduced the risk of stroke (RR = 0.77, 95% CI 0.70–0.85). 113 Furthermore, edaravone dexborneol (Eda.B), a formulation comprising edaravone (30 mg) and (+)-borneol (7.5 mg), was approved by the National Medical Products Administration of China in July 2020 for the clinical treatment of AIS. Studies have shown that Eda.B can reduce NETs levels in serum samples of AIS patients and tissue samples from MCAO mouse models, improve blood–brain barrier (BBB) permeability, and exert neuroprotective effects. 114

Pulmozyme (dornase alfa), a recombinant human deoxyribonuclease I (rhDNase), has received FDA approval to treat cystic fibrosis (CF) clinically. 115 Dornase alfa reduces sputum viscosity by hydrolyzing extracellular DNA released from degenerating neutrophils in the sputum of CF patients, thereby facilitating mucus clearance. 116 Currently, dornase alfa is recommended only for nebulized inhalation. Under this route, it acts primarily at the local pulmonary level, with minimal systemic absorption. Its half-life is approximately 3 to 4 hours in the bronchi and 8 to 11 hours in the lungs, with anticipated metabolism by proteases present in biological fluids. 117 According to the prescribing information of Pulmozyme, the elimination half-life of dornase alfa in human plasma following intravenous injection is approximately 3 to 4 hours. Notably, there have been no reports of hemorrhagic complications associated with dornase alfa treatment to date, which provides a safety foundation for its subsequent clinical application in stroke.

Two ongoing phase II clinical trials are investigating its role in improving early reperfusion rates in AIS. The NETs-target trial (NCT04785066) aims to evaluate the efficacy of intravenous dornase alfa in improving vascular recanalization following thrombectomy in AIS patients. The study plans to enroll adult stroke patients with occlusions in the internal carotid artery, M1 segment, or M2 segment of the middle cerebral artery. After receiving standard therapy (IVT and MT), participants will receive adjunctive intravenous dornase alfa (specific dose has not been disclosed). The EXTEND-IA DNase trial (NCT05203224) seeks to assess whether adjunctive intravenous dornase alfa can improve early reperfusion in large vessel ischemic stroke, and to explore the optimal single bolus dose of dornase alfa. Compared with NETs-target, this study additionally enrolled patients with basilar artery occlusion. Following IVT and/or MT, participants received a single intravenous bolus of dornase alfa at escalating tiers (0.125, 0.25, 0.5, and 1 mg/kg). In both trials, the primary endpoint is the achievement of significant recanalization on post-treatment angiography without symptomatic intracranial hemorrhage. It is worth mentioning that no adverse events have been reported to date in either trial. The findings of these investigations may offer clinical evidence for the use of DNase I in AIS.

### Thrombolytic Benefits of Targeting NETs

Since NETs play a crucial role in thrombolytic resistance, the potential advantages of targeting NETs to improve thrombolytic treatment have been investigated in several studies. Despite the fact that rt-PA by itself did not lower the amount of NETs in the thrombus before thrombectomy, 118 DNase I can effectively degrade NETs by cleaving the DNA scaffold. As a result, DNase I has been proposed as a promising adjuvant in thrombolytic therapy.

To simulate rt-PA-resistant thrombi, Peña-Martínez et al constructed a mouse photothrombotic stroke model to generate fibrin-free thrombi composed primarily of aggregated platelets. They found that DNase I administration alone was sufficient to recanalize occluded vessels, improving stroke outcomes in experimental mice. 119 Additionally, Laridan et al demonstrated that combining DNase I with rt-PA enhanced the lysis of retrieved AIS thrombi. 60 A recent study further showed that the combination of DNase I and rt-PA resulted in a 3-fold increase in thrombolysis efficiency in contrast to rt-PA alone, and the content of histones and DNA in thrombus was related to the lysis sensitivity of DNase I. 120 Considering that erythrocyte-poor thrombi are often resistant to rt-PA, Vandelanotte et al compared the effects of rt-PA combined with DNase I on AIS thrombi with varying erythrocyte content. They found that DNase I overcame the rt-PA resistance of erythrocyte-poor thrombi, but had no additional effect on erythrocyte-rich thrombi, which are already susceptible to rt-PA. 98 Interestingly, it was reported that DNase I alone could not lyse ex vivo AIS thrombi. 61 A subsequent study contradicted this, showing that DNase I was even more effective than rt-PA. This discrepancy may be attributed to two factors: one is the difference in the type of thrombus used (i.e., cryopreserved versus fresh), and the other is the variation in the duration of the ex vivo lysis tests (i.e., 1 hour vs. 4 hours). 119

It is noteworthy that existing studies have demonstrated a synergistic interaction between DNase I and plasmin. Napirei et al showed that DNase I exhibits high activity against protein-free plasmid DNA but is nearly inactive against chromatin (DNA complexed with histones and other proteins). However, upon the addition of serine proteases like plasmin, DNase I can efficiently degrade chromatin. 121 The mechanism involves plasmin removing DNA-binding proteins (primarily histones), thereby exposing the DNA strands and providing DNase I access to its substrate. Additionally, DNase I possesses di-N-glycosylation, which allows it to remain stable and resist proteolytic cleavage in the presence of plasmin. Desilles et al found that DNase I enhances rt-PA-mediated thrombolysis at least partially by promoting fibrin degradation. 122 This suggests that while DNase I may exert a direct thrombolytic effect on thrombi with high NETs burden, it also facilitates indirect fibrinolytic-dependent thrombolysis. This effect is likely due to the degradation of the DNA backbone of NETs, which disrupts thrombus stability and consequently enhances plasmin activity. In summary, the synergy between DNase I and plasmin essentially follows a process of “removing protein barriers first, then cleaving DNA, and finally enhancing plasmin activity.”

Neutrophil stasis in the cerebral capillaries has been demonstrated as a major cause of no reflow after thrombolysis. 123 Notably, the aggregation of NETs has been observed in capillaries, potentially promoting secondary microthrombosis and resulting in microcirculatory dysfunction. 17 Increased risk of hemorrhagic transformation is another defect of rt-PA, which may be because rt-PA increases neutrophils recruitment and induces NETs formation by upregulating LDL receptor-related protein 1 and PAD4, aggravating the inflammation and BBB damage in the lesion. 124 125 126 Notably, NETs have been shown to activate the cGAS-STING pathway, causing type I interferon to be produced, which exacerbates the BBB breakdown and cerebral bleeding brought on by rt-PA. Importantly, targeting NETs with DNase I can reduce BBB damage, improve vascular remodeling, and enhance microcirculation perfusion, thereby mitigating rt-PA-associated cerebral hemorrhage complications. 125

### Other Strategies

In addition to platelet glycoprotein receptors, vWF within the thrombus may serve as another potential therapeutic target. Two promising candidates—N-acetylcysteine and ADAMTS13—have demonstrated significant thrombolytic efficacy in various experimental thrombosis models without inducing an increased risk of hemorrhagic transformation. 127 128 129 Furthermore, targeting endogenous fibrinolytic inhibitors such as PAI-1, thrombin-activatable fibrinolysis inhibitor (TAFI), 130 131 and α2-antiplasmin (α2-AP) may also help overcome rt-PA resistance. 132

## Current Limitations

DNase I holds significant potential for enhancing thrombolysis by degrading NETs. This represents a promising therapeutic approach, particularly given the current limited treatment options. However, it is important to note that the present study has several limitations. First, the thrombi retrieved by MT do not represent the full spectrum of AIS thrombi. Only those thrombi that are either resistant to spontaneous dissolution or successfully retrieved after rt-PA treatment are included in research studies, leaving out thrombi that may be sensitive to rt-PA or resistant to thrombectomy. This limits the generalizability of findings to the broader population of AIS thrombi. Second, most experimental studies have been conducted using in vitro AIS thrombi or synthetic models. It remains unclear whether the mechanisms observed in these controlled settings also apply to the complex in vivo environment of a human stroke. Future research must elucidate the precise function of NETs in the pathological processes of AIS and determine if the findings from laboratory models can be translated to clinical scenarios.

The efficacy and safety of DNase I combined with rt-PA to assist thrombolysis require further clinical studies. The premise for combining DNase I with thrombolytic therapy is that NETs have already formed within the therapeutic time window (4.5 hours). Cha et al established a murine carotid artery occlusion model using FeCl _3_ and collected thrombi at 0.5, 1, 2, 3, 6, and 24 hours post-occlusion. Their findings revealed that NETs within thrombi began to increase as early as 0.5 hour. 52 Similarly, Zhang et al enrolled 60 AIS patients who received intravenous thrombolysis within 4.5 hours of symptom onset. Plasma samples collected before thrombolysis revealed that NETs levels in AIS patients were significantly higher than in healthy controls. 107 Taken together, NETs form within 4.5 hours in at least a proportion of AIS patients. Of course, it is also possible that NETs formation begins beyond 4.5 hours. In such cases, early administration of DNase I could still exert beneficial effects during thrombolysis. First, early co-administration of DNase I may prevent excessive accumulation of NETs, thereby reducing thrombus stability. Second, as previously mentioned, early use of DNase I can mitigate NETs-induced local inflammation and BBB disruption, improve microcirculatory perfusion, and potentially reduce the risk of rt-PA–associated hemorrhagic complications. Finally, DNase I has a relatively long plasma half-life, which further increase the likelihood of early NETs targeting.

Concerns regarding the safety of DNase I primarily include the following points: First, there is uncertainty about whether the degradation of NETs by DNase I, which releases components such as DNA, NE, histones, and other procoagulant substances, might increase the risk of thrombosis. Second, since NETs are part of the host's immune defense system, reducing NETs may elevate the risk of infection in critically ill individuals. Finally, it remains unclear whether DNase I could promote genomic instability in damaged neuronal cells, potentially triggering carcinogenesis. Theoretically, exogenous recombinant DNase I primarily degrades extracellular DNA and does not easily penetrate cell membranes to enter the intracellular environment. Currently, there is no reliable evidence supporting the notion that DNase I induces carcinogenesis. Therefore, long-term and systematic safety monitoring is required in the future to further address these concerns.

## Future Directions

Given that studies have reported higher neutrophil NETs content in cardioembolic thrombi compared with other subtypes and reduced thrombolytic efficacy, 60 103 133 134 135 the NETs' content within thrombi has been proposed as an etiological classifier and therapeutic response indicator. However, thrombus analysis is relatively complex and time-consuming, and is only applicable to patients undergoing thrombectomy. Therefore, the development of rapid and highly specific peripheral blood biomarkers of NETs is essential, as this would assist neurologists in formulating more precise treatment strategies.

Previous studies have demonstrated significantly elevated NETs levels in the plasma of AIS patients. 107 Vallés et al reported that plasma levels of CitH3 and cell-free DNA (cfDNA) were higher in patients with cardioembolic stroke compared with other subtypes. 18 Importantly, Genchi et al found a correlation between NETs content in thrombi and NETs levels in plasma (r = 0.62, *p* ≤ 0.001). 133 Furthermore, Baumann et al observed a negative correlation between thrombus MPO content and plasma MPO–histone complex concentrations (ρ = –0.237, *p* = 0.017), while a positive correlation was found between thrombus DNA–histone-1 complexes and plasma DNase activity (ρ = 0.204; *p* = 0.037), which may reflect endogenous regulatory mechanisms. 136

Nevertheless, caution should be exercised when interpreting these correlations, as further validation in larger cohorts is required. To date, no gold-standard biomarker for NETs has been established. Given the limitations of individual markers, for example, CitH3 only detects PAD4-dependent NETosis and cfDNA lacks specificity, 137 we recommend a combined assessment of CitH3, MPO–DNA complexes, and cfDNA to evaluate NETs levels.

## Conclusion

Given the limitations of current IVT options for AIS, there is an urgent need to identify novel therapeutic targets that can overcome rt-PA resistance mechanisms. NETs are essential to the pathogenic progression of AIS and contribute to thrombolysis resistance in thrombi. The combination of rt-PA and DNase I offers a promising strategy by simultaneously degrading both fibrin and NETs, which improves the efficacy of IVT while reducing the risk of intracranial hemorrhage. This approach holds significant clinical potential for enhancing early reperfusion rates and improving long-term outcomes in AIS patients. However, further clinical studies are necessary to evaluate the efficacy and safety of combining DNase I with rt-PA in thrombolysis.
